# Decreased Sex Hormone-Binding Globulin Indicated Worse Biometric, Lipid, Liver, and Renal Function Parameters in Women with Polycystic Ovary Syndrome

**DOI:** 10.1155/2020/7580218

**Published:** 2020-06-25

**Authors:** Xi Luo, Xin-Ming Yang, Wang-Yu Cai, Hui Chang, Hong-Li Ma, Yan Peng, Xiao-Ke Wu

**Affiliations:** ^1^Heilongjiang University of Chinese Medicine, Harbin, China; ^2^First Affiliated Hospital, Heilongjiang University of Chinese Medicine, Harbin, China; ^3^The Fourth Affiliated Hospital, Zhejiang University School of Medicine, Yiwu, China; ^4^Heilongjiang Province Hospital, Harbin, China

## Abstract

**Objective:**

To investigate the relationships between sex hormone-binding globulin (SHBG) and comprehensive metabolic parameters including biometric, glycemic, lipid, liver, and renal functions of women with polycystic ovary syndrome (PCOS). *Study Design and Methods*. A total of 1000 women diagnosed as PCOS by modified Rotterdam criteria were enrolled in a randomized controlled trial. SHBG and comprehensive metabolic parameters were measured at the baseline visit. Metabolic parameters included biometric parameters, glucose and lipid panels, and liver and renal function parameters. An independent *t*-test and linear regression were performed to investigate the associations between SHBG and metabolic parameters. Logistic regression was used to detect the relationship between SHBG and the presence of metabolic syndrome.

**Results:**

In comparative analyses, PCOS women with lower SHBG levels had higher body mass index, waist circumference, insulin, homeostatic model assessment-insulin resistance (HOMA-IR) index, systolic and diastolic blood pressure, triglycerides, apolipoprotein B (APOB), low-density lipoprotein (LDL), aspartate transferase (AST), alanine transferase (ALT), and blood urea nitrogen (BUN), but lower high-density lipoprotein (HDL) and apolipoprotein A1 (APOA1). In linear regression, SHBG was inversely associated with waist circumference, systolic blood pressure, triglyceride, LDL, APOB, ALT, AST, and BUN but positively associated with HDL and APOA1 after adjusting the BMI. In logistic regression, SHBG is a protective predictor for metabolic syndrome (odds ratio = 0.96; 95% confidence interval: 0.95–0.97). The area under the receiver-operator characteristic curve is 0.732 with a 95% confidence interval of 0.695–0.770. SHBG <26.75 mmol/L is the cutoff point with the best Youden index, which has a sensitivity of 0.656 and specificity of 0.698.

**Conclusions:**

Lower SHBG was associated with worsening biometric, lipid, liver, and renal functions but not glycemic parameters among women with PCOS. SHBG can be used as a tool to screen metabolic syndrome. This trial is registered with NCT01573858 and ChiCTR-TRC-12002081.

## 1. Introduction

Polycystic ovary syndrome (PCOS) is one of the most common reproductive disorders in reproductive-aged women [1]. Apart from reproductive abnormalities such as anovulation and infertility, reliable evidence showed that PCOS is also associated with endocrinologic and metabolic problems. For example, the prevalence of metabolic syndrome (MetS) in PCOS women is higher [2, 3]. PCOS is also more likely to be associated with nonalcoholic fatty liver disease [4], and a hepato-ovarian axis is suggested to explain the relationship between the liver and PCOS [5]. Furthermore, PCOS women may suffer more kidney diseases due to obesity and insulin resistance, which have multiple links with kidney diseases [6, 7]. In clinical practice, it is of great use if we can identify a marker for comprehensive metabolic parameters in women with PCOS.

Sex hormone-binding globulin (SHBG) is a homodimeric transport glycoprotein produced in hepatocytes, binds and regulates sex hormones in the circulation. SHBG is closely related to PCOS due to its regulation on sex hormones. Recently, more evidence indicates the important role of SHBG on metabolic health. Studies have shown that low SHBG levels are predictive of higher risk for developing hypertension [8], type 2 diabetes mellitus (T2DM) [9], and MetS [10] in the general population. Lower serum SHBG is also associated with the elevated serum liver enzyme in men with hepatic steatosis [11]. SHBG is, therefore, thought to play a positive role in metabolism pathways in the human body.

A systematic review shows that the SHBG level of PCOS women is lower than that of the normal control group and SHBG may be a useful biomarker to diagnose PCOS [12]. Furthermore, SHBG has also been found to inversely correlate to MetS in overweight and obese women with PCOS [13]. Therefore, SHBG might be a possible marker for comprehensive metabolic parameters in women with PCOS. However, data on the association between SHBG and metabolic health in women with PCOS are still limited. This article aimed to investigate the associations between SHBG and comprehensive metabolic parameters including biometric, glycemic, lipid, liver, and renal functions in reproductive-aged women with PCOS.

## 2. Methods

### 2.1. Materials and Design

This is a cross-sectional secondary analysis of a randomized controlled trial in mainland China, which has previous publication [14]. The recruitment started from July 2012 to November 2014. There were 1000 women included in 27 tertiary or secondary hospitals across China mainland. The trial was registered on ClinicalTrials.gov (NCT01573858) and chictr.org.cn (ChiCTR-TRC-12002081). All the ethical committees in local sites approved the protocol.

### 2.2. Patients

In this trial, all participants were diagnosed as PCOS by the modified Rotterdam criteria [1], which are also Chinese version PCOS diagnosis criteria [15]. Participants were required to have oligomenorrhea, together with hyperandrogenism (clinical: modified Ferriman–Gallwey hirsutism score >=5 [16], biochemical: serum total testosterone >1.67 mmol/L), polycystic ovaries in ultrasound, or both. All patients signed informed consent. Details can be found in the main article of this trial [14].

### 2.3. Measurements

At the baseline visit, all participants underwent a physical assessment in a standard method by a research assistant. Height, weight, waist circumference, systolic blood pressure (SBP), and diastolic blood pressure (DBP) were measured, and the body mass index (BMI) was calculated by height and weight.

Blood samples were collected at day 3 in the menstrual cycle for each participant at baseline visit. All blood samples were stored at −20°C and shipped back to the core laboratory at Heilongjiang University of Chinese Medicine for measurement. The SHBG level was measured, and metabolic parameters included glucose, insulin, high-density lipoprotein (HDL), low-density lipoprotein (LDL), triglycerides, cholesterol, apolipoprotein A1 (APOA1), apolipoprotein B (APOB), and lipoprotein. Homeostatic model assessment-insulin resistance (HOMA-IR) was calculated by glucose and insulin. Liver function parameters included aspartate transferase (AST), alanine transferase (ALT), and total bilirubin. Renal function parameters included blood urea nitrogen (BUN) and creatinine.

### 2.4. Metabolic Syndrome

MetS was defined by meeting any 3 of the following 5 criteria [1, 14]: (1) waist circumference >88 cm; (2) triglycerides level >1.695 mmol/L; (3) HDL level <1.295 mmol/L; (4) SBP >130 mmHg or DBP >85 mmHg; and (5) fasting glucose level >6.105 mmol/L.

### 2.5. Assay

SHBG was measured by chemiluminescence immunoassay (Siemens). Plasma glucose was measured with a hexokinase assay (Maker), fasting insulin was analyzed with an electrochemiluminescence immunoassay (Roche Diagnostics), HDL and LDL were measured by direct-method assays, and triglyceride and cholesterol were measured by the N-(3-sulfopropyl)-3-methoxy-5-methylaniline method (Wako Diagnostics). Serum APOA1 and APOB levels were determined by the polyethylene glycol-enhanced immunoturbidimetric assay (Maker). AST and ALT were measured with the IFCC method, and total bilirubin was measured with the vanadate oxidation method (Wako Diagnostics). BUN was measured with the UV-GLDH method, and creatinine was measured with the SAO method (Maker).

### 2.6. Statistical Analysis

All data were analyzed using SPSS Statistics (IBM SPSS, Inc., Chicago, IL, USA version 24.0). First, we presented all baseline characteristics in all women as mean ± standard deviation. The independent t-test was used to compare the means between groups with high and low SHBG levels. Then, linear regression was used to detect the relationship between SHBG and each metabolic parameter. Results were presented as regression coefficients, while coefficient >0 indicated positive relationship and coefficient <0 indicated a negative relationship. Logistic regression analysis was performed to explore the association between SHBG and MetS [17, 18]. Results were expressed as odds ratio (OR), and values of >1 indicated an increased chance of the presence of MetS while values of <1 indicated decreased chance of presence of MetS.

The receiver-operating characteristic (ROC) curve of SHBG for MetS was drawn, and the area under the curve was calculated. [19] A value of 0.5 indicates no diagnostic value for MetS, and a value of 1 indicates perfect diagnostic value for MetS. We also selected a cutoff point of SHBG for MetS by the highest Youden index, which is calculated as sensitivity + specificity − 1.

## 3. Results

Table 1 demonstrates the baseline characteristics of all 1000 women with PCOS in our trial. All PCOS women had a mean age of 27.9 ± 3.3 years old, BMI of 24.2 ± 4.3 kg/m^2^, waist circumference of 85.4 cm, SBP of 112.3 mmHg, and DBP of 74.9 mmHg. The mean SHBG level was 42.6 ± 30.7 mmol/L. For the glucose panel, the women had a glucose level of 5.0 mmol/L, insulin level of 98.1 pmol/L, and HOMA-IR level of 3.4. For the lipid panel, results showed an HDL level of 1.3 mmol/L, triglyceride level of 1.6 mmol/L, cholesterol level of 4.7 mmol/L, APOA1 level of 1.5 g/L, APOB level of 0.9 g/L, LDL level of 3.0 mmol/L, and lipoprotein level of 130.1 mmol/L. As for liver and renal functions, the women had an ALT level of 9.0 U/L, AST level of 13.0 U/L, total bilirubin level of 6.4 umol/L, BUN level of 4.4 mmol/L, and creatinine level of 42.9 umol/L.

All 1000 women were divided into low and high SHBG level groups by the median of 33.7 mmol/L (Table 1). PCOS women with lower SHBG level had higher BMI (25.0 vs. 23.8 kg/m^2^, *P* < 0.001), waist circumference (90.2 vs. 80.7 cm, *P* < 0.001), insulin (109.9 vs. 91.8 pmol/L, *P*=0.013), HOMA-IR (3.9 vs. 3.1, *P*=0.032), SBP (113.8 vs. 110.9 mmHg, *P* < 0.001), DBP (75.9 vs. 73.8 mmHg, *P* < 0.001), triglycerides (1.8 vs. 1.4 mmol/L, *P* < 0.001), APOB (1.0 vs. 0.8 g/L, *P* < 0.001), LDL (3.0 vs. 2.9 mmol/L, *P*=0.026), AST (11.1 vs. 6.9 U/L, *P* < 0.001), AST (14.2 vs. 11. U/L, *P* < 0.001), and BUN (4.5 vs. 4.2 mmol/L, *P* < 0.001), but also lower HDL (1.1 vs. 1.4 mmol/L, *P* < 0.001) and APOA1 (1.4 vs. 1.6 g/L, *P* < 0.001).

In linear regression analyses (Table 2), SHBG had inverse associations with BMI (*β* = −0.025, *P* < 0.001), waist circumference (*β* = −0.128, *P* < 0.001), insulin (*β* = −0.288, *P*=0.014), SBP (*β* = −0.026, *P*=0.008), DBP (*β* = −0.017, *P*=0.043), triglycerides (*β* = −0.006, *P* < 0.001), APOB (*β* = −0.002, *P* < 0.001), LDL (*β* = −0.003, *P*=0.004), AST (*β* = −0.032, *P* < 0.001), ALT (*β* = −0.058, *P* < 0.001), and BUN (*β*=−0.005, *P* < 0.001) while had positive associations with HDL (*β* = 0.005, *P* < 0.001) and APOA1 (*β* = 0.004, *P* < 0.001). After adjusting the BMI, the association of DBP and insulin disappeared.

**Figure 1:**
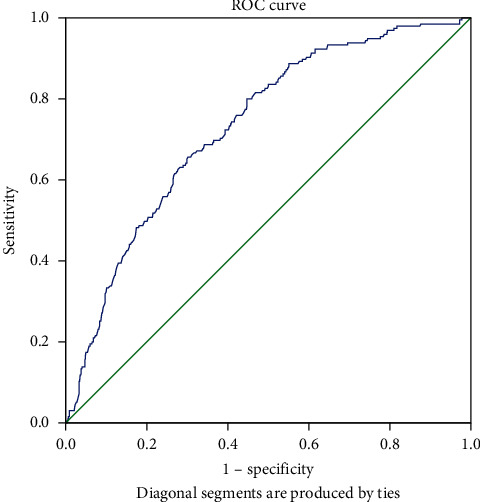
Receiver-operating characteristic curve of SHBG for metabolic syndrome.

Of 1000 women with PCOS, there were 196 women (19.6%) were diagnosed as MetS.  Table 3 shows that SHBG is associated with MetS (OR: 0.96; 95% confidence interval: 0.95–0.97, *P* < 0.001). The ROC curve of SHBG for MetS is demonstrated in Figure 1. The area under the curve was 0.732 with a 95% confidence interval of 0.695–0.770. The SHBG cutoff point of <26.75 mmol/L had the highest Youden index, with a sensitivity of 0.656 and a specificity of 0.698 (Table 4).

## 4. Discussion

In this study, our results showed that the lower SHBG level was associated with worse comprehensive metabolic parameters including biometric, lipid, liver, and renal function but not glycemic parameters among Chinese women with PCOS. Lower SHBG is a marker to reflect worse metabolic health and can be used as a tool to screen MetS for women with PCOS.

The relationship between SHBG and metabolic disorder might be explained by androgen. Lower SHBG usually leads to more free and biologically active androgens in the circulation and might disrupt insulin secretion and pancreatic *β*-cell function and aggravate MetS and insulin resistance in women with PCOS [20–23]. In turn, hyperinsulinemia stimulates testosterone biosynthesis in thecal cells from women with PCOS [24]. Here, we proposed that SHBG could participate in the metabolic pathway through androgen regulation.

Although the clear mechanism between SHBG and insulin resistance is not fully understood, it is found that SHBG could affect glucose transporters and the PI3K/AKT pathway [25, 26]. Recent studies found that lower SHBG was an independent risk factor for the development of type 2 diabetes, and strong genetic evidence existed that SHBG is involved in the etiology of type 2 diabetes [9, 27]. A previous study evaluated SHBG levels in women with PCOS and found an association between low SHBG levels and subsequent development of gestational diabetes mellitus [28]. To the contrary, our study did not find associations between glucose and insulin after adjusting the BMI. A genetic study also found weaker causal effects of SHBG for insulin resistance and diabetes, suggesting that the observational associations are partly confounded rather than conferred directly via circulating SHBG [29]. Our results suggested the association between SHBG and glycemic control dependeding on the BMI, and SHBG cannot reflect insulin resistance for women with PCOS.

Our study also found strong associations between SHBG and blood lipid levels in women with PCOS. Lipid metabolism is linked to PCOS in many ways, and abnormal blood lipid levels can be caused by androgen excess. Previous studies also found that elevated lipoprotein lipase activity was associated with a better lipid profile, and SHBG was observed to have a positive relationship with lipoprotein lipase activity [30]. Since lipid metabolism and production of SHBG both occur in the liver, the association between lipid and SHBG might reflect the change of liver metabolism. Therefore, SHBG might be a good marker to reflect lipid health in women with PCOS.

We also found associations between SHBG and liver and renal function parameters in women with PCOS. The associations between SHBG and androgen might be the reason. Studies have found that hyperandrogenemia contributed to higher blood liver enzyme level in women with PCOS [31, 32]. Studies in humans and animals also suggested that androgens can increase blood pressure and compromise renal function [33]. In addition, SHBG is produced predominantly in hepatocytes and the synthesis of SHBG could be associated with liver function. ALT is known to reflect hepatocellular injury or death. Therefore, hepatocellular injury or death might be associated with increased serum ALT level after the release of ALT from liver cells and also associated with decreased synthesis of SHBG.

Furthermore, more functions related to SHBG have been uncovered in recent years. SHBG receptors are expressed in cell membranes in multiple tissues and reveal different biological effects on cell growth and biochemical endpoints [34]. However, there is still no clear answer to the question of the effect of SHBG on different organs and tissues. We hypothesize that SHBG could affect multiple biological pathways such as lipid metabolism, steroidogenesis, and liver and renal metabolism via SHBG receptors.

For clinical use, we also evaluate the value of SHBG for MetS. MetS is a cluster of unhealthy metabolic components, which has various long-time complications such as type 2 diabetes and cardiovascular diseases. Since the risk of metabolic disorder is rather high in women with PCOS, screening for MetS is important. The comprehensive physical and metabolic panel is lengthy and expensive. Our study supported that SHBG could be used as a screening tool for MetS with decent accuracy. An SHBG level lower than 26.75 mmol/L can predict abnormal metabolic health in Chinese women with PCOS.

There are several strengths of this study. First, the women were recruited from different sites across different geographical regions in China mainland, which increased the generalizability of the cohort. Second, common clinical data were collected in a standardized method, and blood samples were stored in a standard method and measured in a core laboratory. However, data of some women were missing and might decrease the statistical power. This study was also restricted to Chinese Han women, and more studies are needed in different people.

In conclusion, lower SHBG indicated worse metabolic parameters including biometric, lipid, liver, and renal function but not glycemic parameters among women with PCOS. SHBG can also be used as a screening tool for MetS in women with PCOS in future clinical settings.

## Figures and Tables

**Table 1:** Comprehensive metabolic parameters in all PCOS women and divided by low- and high-SHBG groups.

	All women (*n* = 1000)	Low-SHBG group (*n* = 479)	High-SHBG group (*n* = 478)	*P* value
Age (year)	27.9 ± 3.3	28.3 ± 3.3	27.7 ± 3.3	0.006
BMI (kg/m^2^)	24.2 ± 4.3	25.0 ± 4.5	23.8 ± 3.9	<0.001
Waist circumference (cm)	85.4 ± 11.5	90.2 ± 11.0	80.7 ± 9.9	<0.001
SHBG (nmol/L)	42.6 ± 30.7	21.5 ± 6.8	63.8 ± 30.8	<0.001
Glucose (mmol/L)	5.0 ± 1.0	5.1 ± 1.0	5.0 ± 1.0	0.304
Insulin (pmol/L)	98.1 ± 108.5	109.9 ± 130.7	91.8 ± 85.1	0.013
HOMA-IR	3.4 ± 5.5	3.9 ± 7.2	3.1 ± 3.6	0.032
SBP (mmHg)	112.3 ± 9.4	113.8 ± 9.2	110.9 ± 9.3	<0.001
DBP (mmHg)	74.9 ± 7.9	75.9 ± 8.0	73.8 ± 7.6	<0.001
HDL (mmol/L)	1.3 ± 0.4	1.1 ± 0.3	1.4 ± 0.4	<0.001
Triglycerides (mmol/L)	1.6 ± 0.9	1.8 ± 1.0	1.4 ± 0.8	<0.001
Cholesterol (mmol/L)	4.7 ± 1.1	4.8 ± 1.1	4.7 ± 1.1	0.737
APOA1 (g/L)	1.5 ± 0.3	1.4 ± 0.3	1.6 ± 0.3	<0.001
APOB (g/L)	0.9 ± 0.3	1.0 ± 0.3	0.8 ± 0.3	<0.001
LDL (mmol/L)	3.0 ± 0.9	3.0 ± 0.9	2.9 ± 0.9	0.026
Lipoprotein (mg/L)	130.1 ± 102.6	128.8 ± 103.1	129.9 ± 97.6	0.868
ALT (U/L)	9.0 ± 8.6	11.1 ± 10.6	6.9 ± 5.2	<0.001
AST (U/L)	13.0 ± 7.3	14.2 ± 8.3	11.9 ± 6.0	<0.001
Bilirubin (*μ*mol/L)	6.4 ± 3.2	6.2 ± 2.9	6.5 ± 3.4	0.164
BUN (mmol/L)	4.4 ± 1.3	4.5 ± 1.3	4.2 ± 1.2	<0.001
Creatinine (*μ*mol/L)	42.9 ± 10.8	43.3 ± 10.4	42.5 ± 11.0	0.264

SHBG: sex hormone-binding globulin; BMI: body mass index; HOMA-IR: homeostatic model assessment-insulin resistance; SBP: systolic blood pressure; DBP: diastolic blood pressure; HDL: high-density lipoprotein; APOA1: apolipoprotein A1; APOB: apolipoprotein B; LDL: low-density lipoprotein; AST: aspartate transferase; ALT: alanine transferase; BUN: blood urea nitrogen.

**Table 2:** Linear associations between SHBG and metabolic parameters.

	Coefficient *β*	*P* value	Coefficient *β* adjusted for BMI	*P* value
Waist circumference (cm)	−0.128	<0.001	−0.128	<0.001
Glucose (mmol/L)	−0.001	0.385	<0.0001	0.958
Insulin (pmol/L)	−0.288	0.014	−0.086	0.443
HOMA-IR	−0.012	0.053	−0.004	0.550
SBP (mmHg)	−0.026	0.008	−0.024	0.018
DBP (mmHg)	−0.017	0.043	−0.014	0.089
HDL (mmol/L)	0.005	<0.001	0.005	<0.001
Triglycerides (mmol/L)	−0.006	<0.001	−0.006	<0.001
Cholesterol (mmol/L)	0.000	0.788	0.001	0.642
APOA1 (g/L)	0.004	<0.001	0.004	<0.001
APOB (g/L)	−0.002	<0.001	−0.002	<0.001
LDL (mmol/L)	−0.003	0.004	−0.003	0.007
Lipoprotein (mg/L)	−0.002	0.987	−0.022	0.843
ALT (U/L)	−0.058	<0.001	−0.054	<0.001
AST (U/L)	−0.032	<0.001	−0.031	<0.001
Bilirubin (*μ*mol/L)	−0.001	0.713	−0.001	0.849
BUN (mmol/L)	−0.005	<0.001	−0.005	0.001
Creatinine (*μ*mol/L)	0.001	0.913	0.005	0.688

SHBG: sex hormone-binding globulin; BMI: body mass index; HOMA-IR: homeostatic model assessment-insulin resistance; SBP: systolic blood pressure; DBP: diastolic blood pressure; HDL: high-density lipoprotein; APOA1: apolipoprotein A1; APOB: apolipoprotein B; LDL: low-density lipoprotein; AST: aspartate transferase; ALT: alanine transferase; BUN: blood urea nitrogen.

**Table 3:** Logistic regression of SHBG for MetS.

	Odds radio (95% confidence interval)	*P* value
SHBG	0.96 (0.95–0.97)	<0.001

SHBG: sex hormone-binding globulin; MetS: metabolic syndrome.

**Table 4:** Use of the cutoff point 26.75 of SHBG to screen metabolic syndrome.

	SHBG < 26.75	SHBG ≥ 26.75	Total
Metabolic syndrome	128	67	195
Not metabolic syndrome	230	532	762
Total	358	599	957

Sensitivity: 128/195 = 0.656; specificity: 532/762 = 0.698.

## Data Availability

The patient data used to support the findings of this study are available from the corresponding author upon request.
